# The role of clinically relevant intra-abdominal collections after pancreaticoduodenectomy: Clinical impact and predictors. A retrospective analysis from a European tertiary centre

**DOI:** 10.1007/s00423-023-03200-z

**Published:** 2023-12-28

**Authors:** Pablo Lopez, Elizabeth Pando, Nuria Ortega-Torrecilla, Noelia Puertolas, Montse Adell, Nair Fernandes, Daniel Herms, Marta Barros, Laia Blanco, Joaquim Balsells, Ramon Charco

**Affiliations:** 1 Universitat Autónoma de Barcelona, Barcelona, Spain; 2 Department of Hepato-Pancreato-Biliary and Transplant Surgery, Hospital Universitari Vall d’Hebron, Barcelona, Spain

**Keywords:** Postoperative collections, Pancreatectomy complications, Pancreatic fistula, Surgical complications

## Abstract

**Background:**

There is controversial evidence regarding the impact of clinically relevant postoperative intra-abdominal collections (CR-IC) on the clinical course after pancreaticoduodenectomy. C-reactive Protein (CRP) has been validated as a predictor of postoperative pancreatic fistula (POPF). Still, its role in predicting CR-IC has not been studied.

**Methods:**

A retrospective analysis was conducted on patients who underwent PD at a tertiary hospital between October 2012 and October 2017. The incidence of CR-IC, clinically relevant POPF and other complications, as well as mortality and length of hospitalisation, was retrieved. The impact of CR-IR on mortality and major complications was analysed. The serum CRP levels were retrieved on the third and fifth postoperative days (POD3 and POD5), followed by an analysis of sensitivity, specificity, and area under the curve to predict CR-IC using CRP.

**Results:**

One hundred forty patients were enrolled following inclusion and exclusion criteria. The mean age was 66.5 years (15–83). The incidence of CR-IC was 33.7% (47), and CR-POPF was 24.3%. Pancreatic duct diameter ≤ 4 mm was identified as a risk factor related to CR-IC occurrence. The group of patients who developed CR-IC after PD exhibited a higher rate of complications Clavien-Dindo ≥ III compared to patients without CR-IC (40.4% vs 7.5%, *p* < 0.001), as well as other events such as admission to the intensive care unit (25.5% vs 4.3%, *p* < 0.001), the incidence of CR-POPF (66% vs 3.2%, *p* < 0.001), prolonged hospital stay (32 vs 13 days, *p* < 0.001), postoperative haemorrhage (23.4 vs 5.4%, *p* = 0.002), and delayed gastric empty (38.8% vs 11.8%, *p* < 0.001) respectively. Logistic regression analysis identified CR-IC related to POPF as a risk factor for Clavien-Dindo > III: OR = 10.6 (95% CI: 3.90–28.7). No differences in mortality were reported between the CR-IC group and non-CR-IC group. CRP at postoperative day 3 (POD3) > 17.55 mg/dl and CRP at postoperative day 5 (POD5) > 13.46 mg/dl were predictors of CR-IC (AUC: 0.731 and AUC:0.821, respectively).

**Conclusions:**

CR-IC has a significant impact after pancreaticoduodenectomy and is associated with a higher incidence of Clavien-Dindo ≥ III complications. Additionally, CRP levels at POD3 and POD5 play a role in predicting CR-IC. Prospective studies are essential to explore strategies for mitigating the occurrence of CR-IC after PD.

**Supplementary Information:**

The online version contains supplementary material available at 10.1007/s00423-023-03200-z.

## Introduction

Pancreaticoduodenectomy (PD) is one of the most complex surgical procedures in pancreatic surgery. Although mortality rates have decreased over the past decades, PD is still associated with higher rates of postoperative complications ranging from 25 to 65% [1–3].

Postoperative intra-abdominal collections (IC) are a frequent entity and could be related to postoperative complications such as anastomotic leaks (pancreatic fistula, bile leak, gastrojejunal leak), infected haematoma or undetermined intra-abdominal abscess. However, an ongoing debate exists regarding the clinical implications of postoperative intra-abdominal collections, as studies have either associated them with non-relevant complications or found no complications [4]. In the literature, ICs are not systematically reported as a separate entity, making it difficult to determine the true incidence of ICs after PD. Some authors reported an IC incidence between 6 and 11% [3, 5–7]. Postoperative intra-abdominal collections can exhibit a clinically relevant course (CR-IC), requiring antibiotics, interventional drainage, or surgery, and can be associated with secondary systemic complications like systemic inflammatory response syndrome (SIRS) and sepsis [5, 6, 8]. Despite their clinical relevance, there are few reports focused on exploring postoperative intra-abdominal collections and their association with more severe complications (Clavien-Dindo ≥ III) after PD. This knowledge is particularly relevant when dealing with postoperative pancreatic fistula (POPF). Intra-abdominal collections represent a specific manifestation of POPF, resulting in fluid leakage containing active pancreatic enzymes and enteric content. This leakage can lead to severe complications such as sepsis and vascular erosion, ultimately causing life-threatening haemorrhage [6].

The current classification’s definition of POPF severity fails to consider its association with intra-abdominal collections. We hypothesise that the presence of intra-abdominal collections associated with POPF or other complications after PD results in more severe outcomes than their absence. Consequently, pursuing strategies for predicting and mitigating intra-abdominal collections is justified within this context.

Furthermore, biochemical markers had not been assessed as predictors of CR-IC. Several studies suggest that the elevation of the serum C-reactive protein (CRP) levels is correlated with the development of a POPF [9, 10]. Nevertheless, the role of CRP in predicting CR-IC after PD remains unexplored.

This study aims to explore the clinical impact of postoperative clinically relevant intraabdominal collections (CR-IC) associated with POPF and other postoperative complications after PD. It also aims to evaluate the role of postoperative CRP levels in predicting CR-IC.

## Methods

### Study design

A retrospective observational single cohort was conducted at a tertiary referral centre was performed. Adult patients who underwent open pancreaticoduodenectomy for benign or malignant disease were included between January 2012 and December 2017. Patients were divided into the CR-IC group and non-CR-IC based on the presence or absence of clinically relevant intra-abdominal collections (CR-IC).

### Surgical procedure

Until December 2017, all PD procedures were performed using an open approach. A pylorus-preserving PD was performed in all cases. The pancreatojejunal classical duct-to-mucosa anastomosis was carried out using single interrupted PDS 5/0–6/0 sutures with externalised pancreatic duct catheter of 5Fr for cases with a small-calibre pancreatic duct (< 4 mm), or an 8Fr catheter for all other cases. Termino-lateral hepaticojejunostomy was performed for biliary reconstruction, and an ante-colic duodenojejunostomy with a single-layer suture was performed for alimentary tract reconstruction. Two abdominal silicone drains of 19Fr with vacuum aspiration were placed at the end of the intervention: one anterior and one posterior to the pancreaticojejunal anastomosis.

### Postoperative management

Serum C-reactive protein (CRP), complete blood counts, and biochemical laboratory tests were determined on the third and fifth postoperative day or when an abnormal clinical course was observed after surgery.

CRP levels were determined using a standardised scattering turbidimetric assay. The optimal cut-off value for CRP on the third and fifth postoperative days in predicting CR-IC in our cohort was analysed using Youden’s index calculation.

Somatostatin analogues were not routinely administered; they were only used in cases where POPF occurred. Infected POPF was diagnosed through microbiological culture.

Computed tomography (CT) scans were not routinely performed but were carried out in the following situations: based on the clinician’s judgement, when POPF was established and associated with persistent SIRS or a significant deviation from the expected clinical course, and when CR-IC or another type of complication was suspected (duodenojejunal or hepaticojejunal anastomotic leak, intraabdominal bleeding, delayed gastric empty associated with POPF).

The decision to remove the drains was based on the surgeon’s judgment, considering factors such as drain output volume, drain characteristics, and the patient's overall condition. Most frequently, the decision to remove the drain was influenced by the presence of the following: clear drain fluid (serous or serohematic fluid), in the absence of unusual output like intestinal content, blood, or bile), low volume output, lower amylase drain levels, and good clinical evolution (*no signs of SIRS-systemic inflammatory response syndrome*, abdominal pain or distension, nausea or vomiting).

After confirming the absence of a pancreatic fistula, the externalised stent is closed in the early postoperative days and typically removed approximately 5–6 weeks postoperatively at the outpatient clinic.

### Postoperative complications and definitions

The postoperative pancreatic fistula was defined according to the ISGPS classification [11]. Only grade B and C fistulae were considered clinically relevant. Other complications, such as delayed gastric emptying and postoperative haemorrhage, were also defined according to ISGPS [12].

Clinically relevant intraoperative collection (CR-IC) was defined as the presence of an intra-abdominal collection visualised on the CT scan, including any size or shape of intra-abdominal liquid reported by the radiologist; that led to a change in routine clinical practice [13], including any of the follows: antibiotics administration, percutaneous, endoscopic or surgical drainage or other interventional procedure, maintenance of intra-abdominal drainages, use of somatostatin analogues, the need for parenteral nutrition due to ileus secondary to intraabdominal collections, or the development of spontaneous enterocutaneous fistula. When a CR-IC was diagnosed, addressing the underlying cause, if identified, was mandatory.

Suspected infection of IC was assumed when fever (≥ 38 °C), increased leukocytes, CRP, or systemic inflammatory response syndrome appeared. The management of suspected infected IC followed a step-up approach, beginning with intravenous antibiotics, followed by percutaneous or endoscopic drainage, and surgical drainage as the last treatment.

Postoperative complications were categorised according to the Clavien-Dindo classification. Clavien-Dindo ≥ grade III complications were considered "major" complication [14].

### Mortality

Postoperative mortality was considered at any time during the hospitalisation or up to day 90 after surgery.

### Analysis of risk factors for CR-IC development

For the analysis of risk factors for CR-IC and CR-POPF, the following variables were included in the model: (1) pre-existing conditions: age, sex, pre-existing high blood pressure, diabetes mellitus, cardiovascular disease (arrhythmia, myocardial infarction, cardiac valve disease), dyslipidaemia, chronic respiratory disease, chronic renal disease, smoking habit, alcohol habit, chronic pancreatitis as a protective factor, ampullary cancer, (2) intraoperative variables: portal vein (PV) or superior mesenteric vein resection (SMV), intraoperative blood transfusions and small pancreatic duct calibre.

### The impact of CR-IC on Clavien-Dindo ≥ III, intensive care unit, and mortality

An odds ratio analysis was performed to explore the impact of CR-IC and each individual complication on the development of Clavien-Dindo ≥ grade III complications.

We also conducted a logistic regression analysis adjusting for (1) pre-existing conditions, (2) intraoperative variables, and (3) the type of complications in relation to the occurrence of Clavien-Dindo ≥ III complications.

We decided not to include infected collections due to the risk of bias caused by multicollinearity in the regression model, as they are present in most POPF cases. Additionally, this was to avoid overfitting the model because of our small sample size.

### Statistical analysis

The Kolmogorov–Smirnov test was applied to the quantitative variables before parametric tests such as the *T*-student or ANOVA. In cases with a non-normal distribution, non-parametric tests were applied, such as the *U*-Mann Whitney or Kruskal–Wallis test. The Chi-square test and relative risk calculation were used to analyse qualitative data. A *p* value less than or equal to 0.05 was considered statistically significant. Logistic regression analysis was performed to explore the role of potential risk factors for CR-IC and Clavien-Dindo ≥ III complications. The Youden index was calculated for CRP at POD3 and POD5. The area under the curve and the receiving operator characteristics (AUC-ROC) were analysed to evaluate CRP in predicting CR-IC.

### Ethical considerations

Our study was approved by the Ethics Committee of our hospital centre (Code PR(AG)81/2019), following the principles of the Declaration of Helsinki for human research.

## Results

Between October 2012 and October 2017, a total of 140 patients were included in the study. CR-IC and CR-POPF occurred in forty-seven patients (33.5%) and thirty-four (24.3%), respectively.

There were no significant differences in baseline characteristics between the CR-IC and non-CR-IR groups, except for the incidence of chronic respiratory disease in CR-IC group (Table 1).

**Table 1:** Basal characteristics of patients according to the presence of CR-ICs

	All population	Without CR- IC	With CR- IC	*p*-value
	*N* = 140	*n* = 93	*n* = 47	
Age, years (median, range)	66.5 (15–83)	64 (15–83)	69 (43–81)	0.074
Male sex, *n*(%)	77(55%)	49(52.7%)	28(59.6%)	0.439
Pathology, *n*(%)				0.772
Pancreatic adenocarcinoma	70(50%)	49(52.7%)	21(44.7%)	
Ampullary adenocarcinoma	16(11.4%)	10(10–8%)	6(12.8%)	
Neuroendocrine tumour	12(8.6%)	8(8.6%)	4(8.5%)	
Squamous carcinoma	6(4.3%)	2(2.2%)	4(8.5%)	
Chronic pancreatitis	9(6.4%)	8(8.6%)	1(2.1%)	
Cholangiocarcinoma	6(4.3%)	4(4.3%)	2(4.3%)	
Other benign pancreatic disease	10(7.1%)	6(6.5%)	4(8.5%)	
Other malignancy	5(3.6%)	3(3.2%)	2(4.3%)	
IPMN	2(1.4%)	1(1.1%)	1(2.1%)	
GIST tumour	2(1.4%)	1(1.1%)	1(2.1%)	
Duodenal cancer	2(1.4%)	1(1.1%)	1(2.1%)	
Tumoral staging in cancer				0.072
Stage Tis	3(2.7%)	1(1.4%)	2(5.3%)	
StageIA	9(8.0%)	2(2.7%)	7(18.4%)	
StageIB	14(12.5%)	9(12.2%)	5(13.2%)	
StagIIA	22(19.6%)	14(18.9%)	8(21.1%)	
StageIIB	51(45.5%)	38(18.9%)	13(34.2%)	
StageIII	8(7.1%)	6(8.1%)	2(5.3%)	
Stage IV	5(4.5%)	4(5.4%)	1(2.6%)	
Pre-existing conditions, *n*(%)				
Smoking, *n*(%)	33(23.6%)	21(22.6%)	12(25.5%)	0.698
Alcohol, *n*(%)	13(9.3%)	10(10.8%)	3(6.4%)	0.305*
High blood pressure, *n*(%)	46(32.9%)	30(32.3%)	16(34%)	0.832
Diabetes mellitus, *n*(%)	21(15%)	13(14%)	8(17%)	0.634
Cardiovascular disease, *n*(%)	12(8.6%)	6(6.5%)	6(12.8%)	0.173*
Renal chronic disease, *n*(%)	0	0	0	
Respiratory chronic disease, *n*(%)	9(6.4%)	3(3.2%)	6(12.8%)	0.039*
Dyslipemia, *n*(%)	40(28.6%)	24(25.8%)	16(34%)	0.308
Personal history of cancer (%)	24(17.1%)	17(18.3%)	7(14.9%)	0.616

IPMN: Intrapapillar mucinous neoplasm

GIST: Gastrointestinal Stromal Tumour

(*) *Fisher Test* results

CR-IC: Clinically relevant intraabdominal collections

Regarding intraoperative findings, the incidence of small pancreatic duct calibre was higher in the CR-IC group compared to the non-CR-IC group (80.9% vs. 49.5%, respectively, *p* < 0.001). The incidence of PV/VMS resection was similar between the groups (*p* = 0.241).

The incidence of complications was higher in the CR-IC group compared to the non-CR-IC group, including a higher incidence of Clavien-Dindo ≥ III complications and CR-POPF, among others. No differences in ICU admission and mortality between groups were found. Regarding CR-IC management, an invasive procedure (percutaneous or surgical) was necessary for 13 out of 47 (27.6%) patients (Table 2).

**Table 2:** Postoperative outcomes according to the presence of CR-ICs

	All population	Without CR- CI	With CR-CI	*p*-value
	*N* = 140	*n* = 93	*n* = 47	
Pancreatic fistula (POPF)				< 0.001
No pancreatic fistula, *n*(%)	100(71.4%)	84(90.3%)	16(34%)	
Biochemical leak, *n*(%)	6(4.3%)	6(6.5%)	0	
Grade B POPF, *n*(%)	23(16.4%)	3(3.2%)	20(42.6%)	
Grade C POPF, *n*(%)	11(7.9%)	0	11(23.4%)	
*Clinically relevant POPF (B-C), n(%)*	34(24.3%)	3(3.2%)	31(66%)	< 0.001
Delayed gastric empty, *n*(%)	29(20.7%)	11(11.8%)	18(38.3%)	< 0.001
Bile leak, *n*(%)	10(7.1%)	3(3.2%)	7(14.9%)	0.017*
Enterocutaneous fistula, *n*(%)	3(2.1%)	0	3(6.4%)	0.036*
Post-operative haemorrhage, *n*(%)	16(11.4%)	5(5.4%)	11(23.4%)	0.002
Intraoperative transfusions, median ± SD	0 (0–1)	0 (1–1)	0 (0–1)	0.759
Postoperative transfusions, median ± SD	0 (0–22)	0 (0–6)	1 (0–22)	< 0.001
Collections requiring percutaneous drainage, *n*(%)	4(2.9%)	0	7(14.9%)	< 0.001*
Collections requiring surgical debridement, *n*(%)	7(5.0%)	0	4(8.5%)	< 0.001*
Positive cultures in drainages, *n*(%)	37(26.4%)	4(4.3%)	33(70.2%)	< 0.001
Clavien-Dindo grade of complications, *n*(%)				< 0.001
No complications	48(34.3%)	48(51–6%)	0	
I	7(5%)	7(7.5%)	0	
II	59(42.1%)	31(33.3%)	28(59.6%)	
III	12(8.6%)	4(4.3%)	8(17%)	
IV	10(7.1%)	1(1.1%)	9(19.1%)	
V	4(2.9%)	2(2.2%)	2(4.3%)	
Clavien-Dindo ≥ grade III, *n*(%)	26(18.6%)	7(7.5%)	19(40.4%)	< 0.001
Hospital length stay, days (median, range)	17 (8–189)	13 (8–189)	32 (10–184)	< 0.001
ICU admission, *n*(%)	16(11.4%)	4(4.3%)	12(25.5%)	< 0.001
Mortality, *n*(%)	4(2.9%)	2(2.2%)	2(4.3%)	0.412*

*POPF* postoperative pancreatic fistula, *CR-IC* clinically relevant intraabdominal collections, *ICU* intensive care unit

^*^Fisher test, otherwise: *Chi*-*square *test

Among patients with CR-POPF, thirty-one of them (91%) had associated intra-abdominal collections (sixteen of them presented Clavien-Dindo ≥ III complications).

### Drain removal

We assessed the timing of drain removal based on the previously explained criteria and the occurrence of CR-POPF. We identified the date of drain removal date in 117 clinical records. The rate of CR-POPF was 5.1% in patients in whom the drains were removed within the first ten days after surgery, compared to 51.3% CR-POPF in patients in whom the drains were removed beyond the tenth postoperative day (*p* < 0.001).

When considering patients whose drains were removed within the first five postoperative days, the rate of CR-POPF was 0%. This suggests that the clinical criteria for drain removal were accurate in most cases.

### Risk factors related to CR-CI and CR-POPF

Patients with a small pancreatic duct calibre (≤ 4 mm) had a higher incidence of CR-CI and CR-POPF compared with patients with a pancreatic duct calibre > 4 mm (45.2% vs 19.1%, *p* < 0.001 and 35.7% vs 11.8%, *p* =  < 0.001 respectively).

After logistic regression analysis, we found that a pancreatic duct calibre ≤ 4 mm was the only risk factor related to CR-CI development (OR: 4.3, CI 95%: 1.9–9.9).

For CR-POPF occurrence, pancreatic duct calibre < 4 mm and smoking habit were identified as risk factors (OR: 7.7, CI95%: 2.5–24.3 and OR 3.8, CI95%:1.2–7.7 respectively).

### Clavien-Dindo ≥ III complications

Complications related to the occurrence of Clavien-Dindo ≥ III complications included CR-IC in general (OR: 8.3), CR-POPF (OR: 8.5), CR-POPF associated with CR-IC (OR: 10.6), postoperative haemorrhage and bile leak. The odds ratio probability for Clavien-Dindo ≥ III and ICU admission increased when complications were associated with CR-IC (Table 3).

**Table 3:** The impact of CR-IC on major outcomes

	Complications related to Clavien-Dindo III complications				ICU admission			
	No (*n* = 114)	Yes (*n* = 26)	*p*-value	OR (CI95%)	No (*n* = 114)	Yes (*n* = 26)	*p*-value	OR (CI95%)
Events								
CR-IC in general	28(24.6%)	19(73.1%)	< 0.001	8.3 (3.2–21.9)	35(28.2%)	12(75%)	< 0.001	7.6 (2.3–25.3)
CR-POPF (grade B-C)	18(15.8%)	16(61.5%)	< 0.001	8.5 (3.3–21.8)	23(18.5%)	11(68.8%)	< 0.001	9.7 (3.0–30.5)
CR-POPF + IC	15(48.4%)	16(61.5%)	< 0.001	10.6 (4.0–27.5)	20(16.1%)	11(68.8%)	< 0.001	11.4 (3.6–36.5)
Delayed gastric emptying	20(17.5%)	9(34.6%)	0.053	–	24(19.4%)	5(31.3%)	0.213	–
Delayed gastric emptying + IC	11(9.6%)	6(23.1%)	0.660	–	13(10.5%)	4(25%)	0,107	–
Hemorraghe in general	8(7%)	8(30.8%)	0.002	5.9 (2.0–17.7)	11(8.9%)	5(31.3%)	0.021	4.7 (1.4–15.9)
Hemorraghe + IC	3(2.6%)	8(30.8%)	< 0.001	16.4 (4.0–67.8)	6(4.8%)	5(31.3%)	0,003	8.9 (2.3–34)
Bile leak	2(1.8%)	8(30.8%)	< 0.001	24.9 (4.9–126.7)	5(4%)	5(31.3%)	0.002	10.8 (2.7–43.2)
Bile leak + IC	1(0.9%)	6(23.1%)	< 0.001	33.9 (3.8–296.8)	3(2.4%)	4(25.0%)	0,003	13.4 (2.7–62.7)

*POPF* postoperative pancreatic fistula, *CR-IC* clinically relevant intraabdominal collections

Mortality occurred in four patients of the entire cohort (2.9%); from these, two cases presented CR-IC secondary to complicated CR-POPF and secondary intra-abdominal haemorrhage; one due to cardiovascular arrhythmia, and the last case developed a rupture/dissection of the common hepatic artery.

We tested logistic regression models, initially including pre-existing conditions and intraoperative variables and gradually introducing complications, starting with CR-IC and adding other complications. Both CR-IC and CR-POPF behaved as related factors. To avoid multicollinearity, we decided to group CR-IC as either related to CR-POPF or unrelated to CR-POPF, resulting in a significant odds ratio that remained stable across various model tests. The logistic regression analysis confirmed that CR-IC related to POPF was a risk factor for major complications in the models performed (Table 4).

**Table 4:** Logistic regression of risk factors related to Clavien-Dindo ≥ III complications

Model 1 (*)	**OR (CI95%)**	***p***
Clinically relevant intraabdominal collections-POPF related	10.6 (3.90–28.7)	< 0.001
Model 2 (*)	**OR (CI95%)**	***p***
Cardiovascular disease	5.4 (1.06–27.18)	0.042
Clinically relevant intraabdominal collections-POPF related	10.6 (3.36–33.65)	< 0.001
Bile leak	21.8 (3.53–134.61)	< 0.001

^*^Model 1 included: Pre-existing conditions, intraoperative variables and clinically relevant intraabdominal collections-POPF related

^**^Model 2 included: Pre-existing conditions, intraoperative variables and clinically relevant intraabdominal collections-POPF related, bile leak, enterocutaneous fistula, post operative haemorrhage, delayed gastric emptying. POPF: post operative pancreatic fistula

### CR-IC prediction sub-analysis

CRP levels at POD3 were available in 103 cases, CRP levels at POD5 were available in 123 cases.

A sub-analysis of the potential predictors of CR-IC was performed on 95 patients who completed the CRP determination on POD3 and POD5.

Patients were divided into the positive or negative CRP group according to the Youden's index cut-off, calculated for CRP and CR-IC occurrence, which were 17.55 mg/dl and 13.46 mg/dl at POD3 and POD5, respectively.

No significant differences in the baseline characteristics were found between groups (CRP-POD5 > 13.55 mg/dl and CRP-POD5 > 17.55 mg/dl), except for a higher incidence of pre-existing high blood pressure in the group of positive CRP at POD3 (Supplementary Table 1).

### Intraoperative characteristics

A positive CRP at POD3 and POD5 was significantly associated with a small pancreatic duct calibre (≤ 4 mm) compared with negative CRP groups (*p* = 0.012 and p = 0.001, respectively) (Supplementary Table 2).

### Analysis of C-reactive protein in predicting CR-IC and complications

A positive CRP at POD3 and POD5 was related to a higher incidence of CR-IC and other postoperative outcomes, such as CR-POPF, infected collections, positive microbiological culture in drainages, Clavien-Dindo ≥ III complications, ICU stay, postoperative transfusions, and hospital length, compared to the negative groups (Supplementary Table 3).

**Fig. 1:**
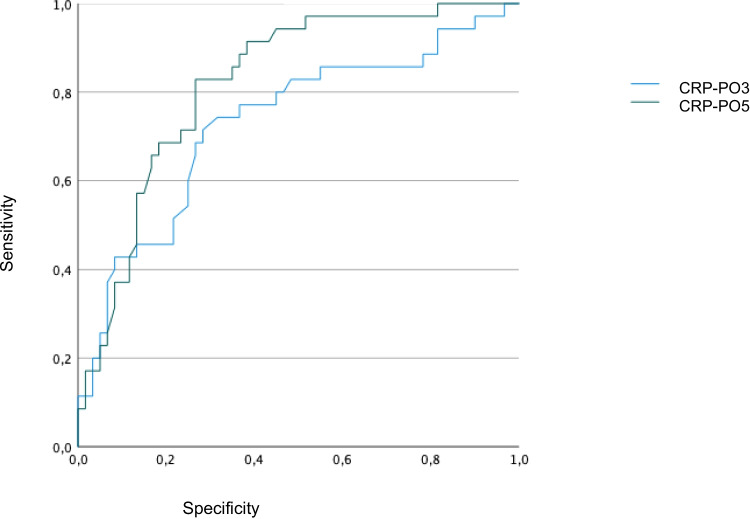
Receiver operating characteristic (ROC) and area under the curve (AUC) of CRP-PO3 and CRP-PO5 for clinically relevant intrabdominal collections

Sensitivity and specificity values for CRP-POD3 in predicting CR-IC was 77.1% (CI95%: 61–-87%) and 61.7% (CI95%:49–72.9%) and for CRP-POD5: 82.9% (CI95%: 67.3–91.9%) and 73.3% (CI95%: 61–82.9%) respectively (Supplementary Table 4). The analysis of AUC-ROC in predicting CR-IC showed an AUC of 0.731 (0.622–0.84) for CR-POD3 and an AUC 0.821 (0.736–0.906) for CRPOD5 (Fig. 1). There were no significant differences when comparing both AUCs using the DeLong test (*p* = 0.062).

### CR-IC evolution and CRP values

**Fig. 2:**
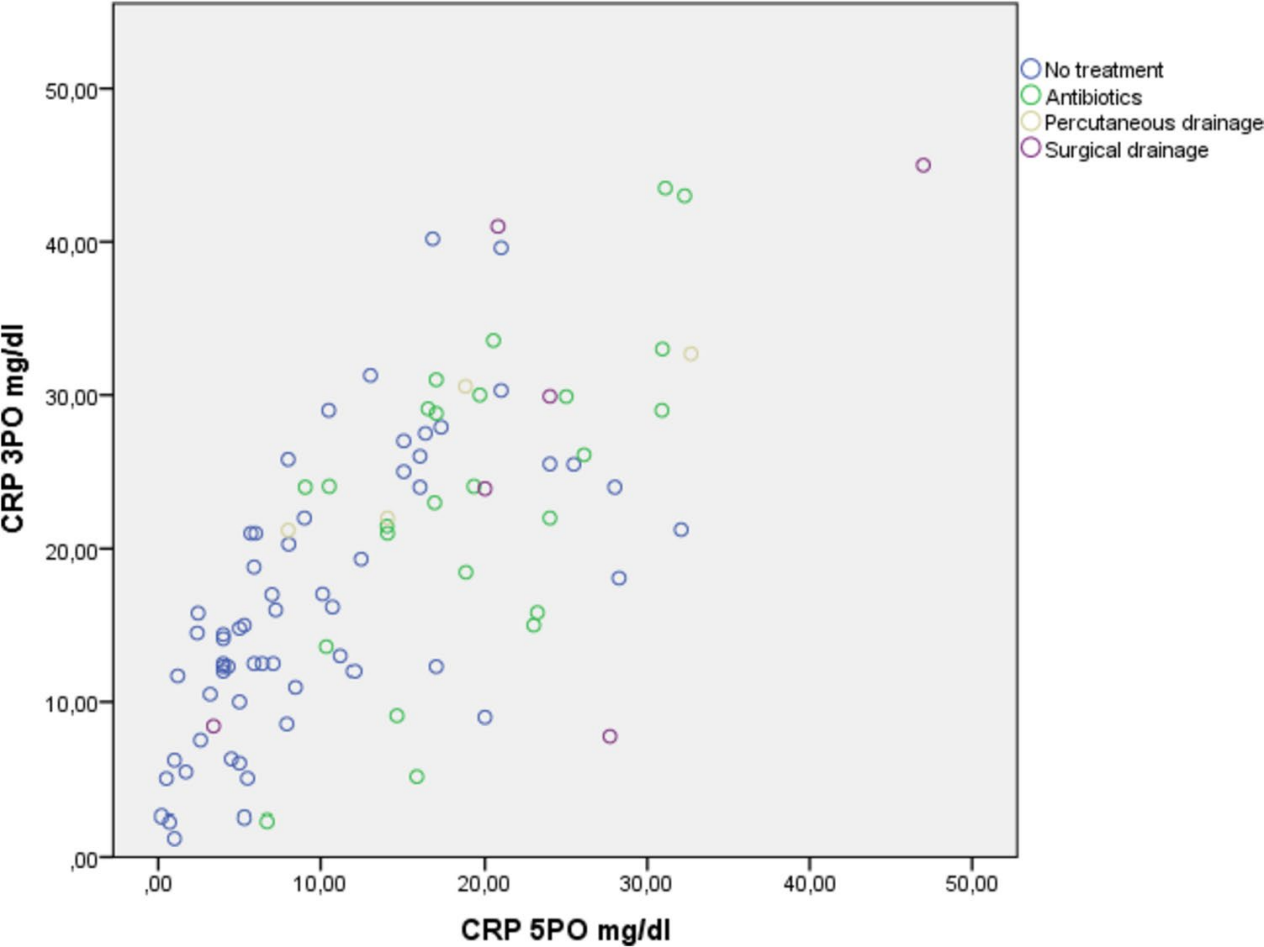
CRP-PO3 and CRP-PO5 performance according to the type of treatment over CR-ICs

Serum values of CRP at POD3 and POD5 were relatively higher when CR-IC was related to CR-POPF compared to bile leak and haemorrhage (Supplementary Table 5). Similarly, serum values of CRP at POD3 and POD5 were higher when an interventional procedure (surgical or percutaneous drainage) was needed (Fig. 2).

## Discussion

### CR-IC: an old entity playing a crucial role after PD

This study explores the clinical impact of CR-IC after pancreaticoduodenectomy (PD) and its prediction using serum CRP. Our results establish CR-IC as a significant event after PD, closely associated with Clavien-Dindo ≥ III, comparable to CR-POPF. Additionally, the presence of CR-IC aggravates the postoperative clinical outcomes when it coexists with other classical complications (POPF, bile leak, haemorrhage). This is one of the few studies reporting CR-IC as a separate entity and describing its negative impact after PD.

Furthermore, we found that serum CRP-POD3 > 17.55 mg/dl and CRP-POD5 > 13.46 mg/dl were associated with CR-IC development in patients after PD. In our cohort, CRP > 13.46 mg/dl at POD5 showed good performance as a predictor test of CR-IC and CR-POPF, with good sensitivity, specificity, and AUC values (0.82).

In the last two decades, postoperative abdominal collections have been systematically reported separately from classical complications such as CR-POPF, bile leak or infected haematoma. However, these last complications are frequently associated with intra-abdominal collections and consequently, the real incidence of "intra-abdominal collections" remains unknown [3, 5, 7].

Recently, some authors reported the incidence of intra-abdominal collections after PD to be around 50–60% (clinically relevant or not). These studies found a relationship between intra-abdominal collections in general and the development of clinically relevant POPF [15, 16]. However, the clinical impact of IC remains controversial, found to be related only to “minor” complications (Clavien-Dindo ≤ IIIa) [4], while some publications reported an increased rate of Clavien-Dindo ≥ III complications (55% vs 35%, *p* = 0.033), as well as the rate of CR-POPF when intra-abdominal collections were present (29% vs 11%, *p* = 0.020) [6].

The relevance of reporting CR-IC relies on the following hypothesis: a poorly drained collection secondary to a surgical complication (POPF, bile leak, haematoma, intestinal fistulae) will worsen the clinical prognosis of the complications itself, increasing the probability of interventional or surgical procedures, sepsis, or organ failure, contrary to a well-drained complication. Even in a scenario of distal pancreatectomy, in which there is no risk of leakage from enteric anastomosis, a recent report indicates that patients with CR-POPF collections had an increased incidence of other pancreatic surgery-specific complications.

The clinical relevance of POPF associated with an intra-abdominal collection (IC) varies according to the type of treatment or the development of systemic complications. For POPF-associating IC, a percutaneous (Clavien-Dindo IIIa) or surgical drainage (Clavien-Dindo IIIb) could be necessary. In the presence of organ failure, a Clavien-Dindo IVa or IVb will occur depending on single or multiple organ failure.

On the other hand, a POPF without an IC will probably have an indolent clinical course, needing antibiotics or the maintenance of surgical drains (also considered a grade B, but a Clavien-Dindo grade II). Considering that, it is still being debated whether grade B-POPF has to be redefined, approaching a most realistic classification to differentiate whether a grade B POPF will be more deleterious inside the same classification group [17]. Our study provides new data regarding the impact of CR-IC in aggravating a CR-POPF clinical outcome, increasing the risk of Clavien-Dindo ≥ III complications and ICU admission when both entities coexist.

In that line, if relevant postoperative collections are the key in the clinical evolution after PD, mainly when POPF occurs, we hypothesise that “well-positioned drains” in a high-risk pancreatic anastomosis (PA) would have an indolent clinical course, compared to a “non” well-positioned drains around (PA), with the secondary development of an intra-abdominal abscess or diffuse peritonitis, as suggested by the previous studies [18].

Regarding whether to drain a pancreatic anastomosis correctly, a prospective study conducted by the Verona Group analysed the phenomenon of “drains dislocation” following pancreatic surgical resections (both PD and DP) [19]. The study revealed that drain dislocation was a frequent occurrence by POD3 and did not significantly impact postoperative complications. In fact, it observed a lower incidence of POPF and major complications. However, the study did not assess the precise location of drains in relation to the pancreatic anastomosis or pancreatic stump in the group of “non-dislocated” drains. Therefore, further evidence is required to elucidate the role of drain placement, the number of drains, and the distance from the anastomosis after PD and their influence on the development of clinically relevant POPF collections.

The use of routine intra-abdominal drains after pancreatectomy is still a debated topic. Previous studies concluded that *routine* intra-abdominal drainage strategies did not offer advantages over *non-routine use*; however, no stratification by high-risk pancreas was performed, and the sample size includes distal pancreatectomy in some studies [20–22]. Although those classical studies advocating a non-routine use of drainages did not perform a routine radiological computed tomography scan during the postoperative period, the impact of *mispositioned* drains in the “routine use” group was not reported [20]. A recent meta-analysis found that leaving routine drains after a PD with a *high pancreatic fistula risk* is beneficial [23, 24].

Additionally, well-designed randomised control trials have concluded that *non-routine* intra-abdominal drainage after PD increased mortality (12% vs. 3%); mortality was significantly higher in patients with risk factors for POPF (small pancreatic duct calibre, soft pancreas texture), confirming that non-routine drains after PD would not be recommended in high-risk pancreatic anastomosis [25]. Our cohort had 64.2% of patients with small pancreatic duct calibre (≤ 4 mm). While all patients received routine postoperative drains, 45.9% developed CR-IC. One limitation of this retrospective study is that we did not assess the exact position of drains and their relation to the development of CR-IC and other major complications.

### CR-IC prediction

C-reactive protein has been validated as an anastomotic dehiscence and intra-abdominal infection predictor in fields other than pancreatic surgery, such as colorectal and oesophageal surgery [26, 27]. Thereby, concomitant collections or infections may explain the release of acute-phase reactant, which could be related to an unfavourable clinical course. According to previous investigations, CRP values at POD3 or POD5 have helped predict clinically relevant POPF. (grade B-C according to ISGPS) [28–33]. Our study among the few in the literature to demonstrate that CRP could also help identify a CR-IC, one of the most relevant complications in our cohort. C-reactive protein at POD5 showed a slight superiority over CRP-POD3 in predicting CR-IC, but no statistical differences were found after applying the *De Long test*. Interestingly, CRP-POD3 and CRP-POD5 values tended to be higher when associated with the need for interventional procedures or associated with the development of POPF when an intra-abdominal collection occurred.

C-reactive protein is a widely available and easily measured method. The findings of this study could help guide decisions to perform an early CT scan to identify CR-IC requiring drainage or to initiate empirical antibiotic treatment.

One of the biases of this study is its retrospective nature, which resulted in the loss of some relevant data, such as pancreatic texture in the surgical report and intraoperative blood loss. Another bias is the unknown incidence of all intra-abdominal collections, including those not clinically relevant. Additionally, the incidence of “well-positioned” drainages and their relationship with outcomes is unknown. However, this study reflects usual clinical practice, considering only clinically relevant intra-abdominal collections, where any deviation from the ordinary postoperative clinical course is present.

## Conclusions

This study has identified CR-IC as a significant complication following pancreaticoduodenectomy, mainly when associated with POPF. Additionally, CRP levels at POD3 and POD5 play a role in predicting CR-IC and CR-POPF. Prospective studies are necessary to investigate methods to reduce the occurrence of CR-IC after PD.

### Supplementary Information

Below is the link to the electronic supplementary material.

Supplementary file1 (DOCX 25 KB)

Supplementary file2 (DOCX 20 KB)

Supplementary file3 (DOCX 23 KB)

Supplementary file4 (DOCX 20 KB)

Supplementary file5 (DOCX 21 KB)

## Data Availability

The authors confirm the accessibility of the data substantiating the results presented in this study, which can be accessed in the main manuscript and its Supplementary Material by requesting the data at https://dataverse.csuc.cat/
