# Supplementary material for: The role of clinically relevant intra-abdominal collections after pancreaticoduodenectomy: Clinical impact and predictors. A retrospective analysis from a European tertiary centre
Source: Langenbecks Arch Surg. 2023 Dec 28;409(1):21. doi: 10.1007/s00423-023-03200-z (PMC10752846; doi:10.1007/s00423-023-03200-z)
Supplement: Supplementary file 1 — Supplementary file1 (DOCX 25 KB) [file 423_2023_3200_MOESM1_ESM.docx]

**Supplementary Table 1. Basal population characteristics according to positive or negative C-reactive protein values at PO3 and PO5.**

|  | **All population**  N=90 | **CRP positive at PO3 (>17.55mg/dl)**  n=50 | **CRP negative at PO3 (<17.55mg/dl)**  n=45 | *p-value* | **CRP positive at PO5 (>13.46mg/dl)**  n=45 | **CRP negative at PO5 (>13.46mg/dl)**  n=50 | *p-value* |
| --- | --- | --- | --- | --- | --- | --- | --- |
| Age, years (mean range) | 53 (30-83) | 50 | 45.7 | 0.449 | 52.78 | 43.70 | 0.109 |
| Sex, male (%) | 41 (43.2%) | 29 (64.4%) | 25 (50.0%) | 0.156 | 30 (60%) | 24 (53.3%) | 0.512 |
| **Pathology, n(%)** |  |  |  | 0.446 |  |  | 0.619 |
| Pancreatic adenocarcinoma | 45(47.4%) | 24(53.3%) | 21(42%) |  | 25(50%) | 20(44.4%) |  |
| Ampullary adenocarcinoma | 10 (10.5%) | 5(11.1%) | 5(10%) |  | 5 (10%) | 5 (11.1%) |  |
| Neuroendocrine tumour | 8 (8.4%) | 3(6.7%) | 5(10.0%) |  | 4 (8%) | 4 (8.9%) |  |
| Squamous carcinoma | 6(6.3%) | 0 | 6(12%) |  | 2(4%) | 4(8.9%) |  |
| Chronic pancreatitis | 8(10%) | 4(8.9%) | 4(8%) |  | 5(10%) | 3(6.7%) |  |
| Cholangiocarcinoma | 4(4.2%) | 1(2.2%) | 3(6%) |  | 1(2%) | 3(6.7%) |  |
| Other benign pancreatic diseases | 7(7.4%) | 4(8.9%) | 3(6%) |  | 5(10%) | 2(4.4%) |  |
| Other malignancy | 3(3.2%) | 2(4.4%) | 1(2%) |  | 2(4%) | 1(2.2%) |  |
| IPMN | 1(1.1%) | 1(2.2%) | 0 |  | 1(2%) | 0 |  |
| GIST tumour | 2(2.1%) | 1(2.2%) | 1(2%) |  | 0 | 2(4.4%) |  |
| Duodenal cancer | 1(1.1%) | 0 | 1(2%) |  | 0 | 1(2.2%) |  |
| **Tumoral Stage, n(%)** |  |  |  | 0.319 |  |  | 0.189 |
| Tis | 3(4.1%) | 1(3%) | 2(4.9%) |  | 1(2.7%) | 2(5.4%) |  |
| Stage IA | 6(8.1%) | 1(3%) | 5(12.2%) |  | 1(2.7%) | 5(13.3%) |  |
| Stage IB | 10(13.5%) | 2(6.1%) | 8(19.5%) |  | 4(10.8%) | 6(16.2%) |  |
| Stage IIA | 17(23%) | 8(24.2%) | 9(22%) |  | 7(18.9%) | 10(27%) |  |
| Stage IIB | 31(41.9%) | 18(54.5%) | 13(31.7%) |  | 18(48.6%) | 13(35.1%) |  |
| Stage III | 4(5.4%) | 2(6.1%) | 2(4.9%) |  | 3(8.1%) | 1(2.7%) |  |
| Stage IV | 3(4.1%) | 1(3%) | 2(4.9%) |  | 3(8.1%) | 0 (0%) |  |
| **Pre-existing conditions (%)** |  |  |  |  |  |  |  |
| Smoking, n(%) | 25 (26.3%) | 12 (26.70%) | 13 (26.0%) | 0.941 | 13 ( 26%) | 12 (26.7%) | 0.941 |
| Alcohol, n(%) | 9 (9.5%) | 5 (11.1%) | 4 (8.0%) | 0.433 | 5 (10%) | 4 (8.9%) | 0.567* |
| High blood pressure, n(%) | 29 ( 30.5%) | 13 (28.90%) | 16 (32.0%) | 0.742 | 15 (30%) | 14 (31.1%) | 0.907 |
| Diabetes mellitus, n(%) | 13 (13.7%) | 2 (4.4%) | 11 (22.0%) | 0.013 | 5 (10%) | 8 (17.8%) | 0.271 |
| Cardiopathy, n(%) | 7 (7.4%) | 2 (4.4%) | 5 (10%) | 0.264 | 2 (4%) | 5 (11.15) | 0.176* |
| Pulmonary obstructive disease, n(%) | 7 (7.4 %) | 3 (6.7%) | 4 (8%) | 0.559 | 2 (4%) | 5 (11.1%) | 0.176* |
| Chronic pancreatitis, n(%) | 8(8.4%) | 4(8.9%) | 4(8%) | 0.582* | 5(10%) | 3(6.7%) | 0.418* |
| Dyslipidemia, n(%) | 28 (29.5 %) | 13 (28.90%) | 15 (30%) | 0.906 | 12 (24%) | 16 (35.6%) | 0.217 |
| Previous history of cancer (%) | 14 (14.7%) | 6 (13.3%) | 8 (16.0%) | 0.714 | 5 (10%) | 9 (20%) | 0.170 |
|  |  |  |  |  |  |  |  |

*IPMN: Intraductal papillary mucinous neoplasm, GIST: Gastrointestinal stromal tumour. *Fisher test.*
