# Supplementary material for: The role of clinically relevant intra-abdominal collections after pancreaticoduodenectomy: Clinical impact and predictors. A retrospective analysis from a European tertiary centre
Source: Langenbecks Arch Surg. 2023 Dec 28;409(1):21. doi: 10.1007/s00423-023-03200-z (PMC10752846; doi:10.1007/s00423-023-03200-z)
Supplement: Supplementary file 2 — Supplementary file2 (DOCX 20 KB) [file 423_2023_3200_MOESM2_ESM.docx]

|  | **All population**  N=95 | **CRP positive at PO3 (>17.55mg/dl)**  n=50 | **CRP negative at PO3 (<17.55mg/dl)**  n=45 | ***p-value*** | **CRP positive at PO5 (>13.55mg/dl)**  n=45 | **CRP negative at PO5 (>13.55mg/dl)**  n=50 | ***p-value*** |
| --- | --- | --- | --- | --- | --- | --- | --- |
| Wirsung duct < 4mm, n (%) | 61 (64.2%) | 25 (55.6%) | 36 (72.0%) | 0.095 | 25 (50%) | 36 (80%) | 0.002 |
| Vein resection, n (%) | 16 (16.8%) | 8 (17.8%) | 8 (16.0%) | 0.817 | 12 (24%) | 4 (8.9%) | 0.049 |
| Amylase drain 3 PO, U/l, mean +/- SD | 57.69 +/- 104.4 | 32.95 +/- 68.5 | 79.91 +/- 125 | 0.026 | 32.34 +/- 87.1 | 85.93 +/- 115.3 | 0.014 |
| UPL Serum Amylase 3PO (>54 U/l), n (%) | 27 (28.4%) | 7 (15.6%) | 20 (40%) | 0.008 | 5 (10.0%) | 22 (48.9%) | <0.001 |

**Supplementary Table 2. Intraoperative findings according to positive or negative C-reactive protein values at PO3 and PO5.**
