# Supplementary material for: The role of clinically relevant intra-abdominal collections after pancreaticoduodenectomy: Clinical impact and predictors. A retrospective analysis from a European tertiary centre
Source: Langenbecks Arch Surg. 2023 Dec 28;409(1):21. doi: 10.1007/s00423-023-03200-z (PMC10752846; doi:10.1007/s00423-023-03200-z)
Supplement: Supplementary file 3 — Supplementary file3 (DOCX 23 KB) [file 423_2023_3200_MOESM3_ESM.docx]

**Supplementary Table 3. Clinical outcomes according to positive or negative C-reactive protein values at PO3 and PO5.**

| **Outcomes** | **All population** | **CRP positive at PO3 (>17.55mg/dl)** | **CRP negative at PO3 (<17.55mg/dl)** | ***p-value*** | **CRP positive at PO5 (>13.55mg/dl)** | **CRP negative at PO5 (>13.55mg/dl)** | ***p-value*** |
| --- | --- | --- | --- | --- | --- | --- | --- |
|  | N=95 | n=50 | n=45 |  | n=45 | n=50 |  |
|  |  |  |  |  |  |  |  |
| Clinically relevant intraabdominal collections (CR-IC), n (%) | 35 (36.8%) | 8 (17.8%) | 27 (54.0%) | <0.001 | 6 (12%) | 29 (64.4%) | <0.001 |
| Clinically Relevant POPF, n (%) | 27 (28.4%) | 7 (15.6%) | 20 (40%) | 0.008 | 3 (6%) | 24 (53.3%) | <0.001 |
| Bile leak, n (%) | 9 (9.5%) | 3 (6.7%) | 6 (12.0%) | 0.299 | 3 (6%) | 6 (13.3%) | 0.193* |
| Delayed gastric empty grade B-C, n (%) | 20 (21.1%) | 10 (22.2%) | 10 (20.0%) | 0.791 | 9 (18%) | 11 (24.4%) | 0.442 |
| enterocutaneous fistula, n (%) | 2 (2.1%) | 0 (0%) | 2 (4%) | 0.274 | 1 (2%) | 1 (2%) | 0.726* |
| Post operative haemorrhage grade B-C, n (%) | 15 (15.8%) | 5 (11.1%) | 10 (20.0%) | 0.235 | 5 (10%) | 10 (22.2%) | 0.103 |
| ICU admission, n (%) | 12 (12.6%) | 2 (4.4%) | 10 (20.0%) | 0.023 | 2 (4%) | 10 (22.2%) | 0.008 |
| CR-IC requiring percutaneous drainage, n (%) | 4(4.2%) | 0 (0%) | 4 (8%) | 0.072 | 1 (2.0%) | 3 (6.7%) | 0.27 |
| CR-IC requiring surgical debridement, n (%) | 6(6.3%) | 2 (4.4%) | 4 (8%) | 0.39 | 1 (2%) | 5 (11.1%) | 0.08 |
| Positive bacterial cultures in drainages, n (%) | 30 (31.6%) | 5 (11.1%) | 25 (50%) | <0.001 | 3 (10%) | 27 (90%) | <0.001 |
| **Clavien-Dindo complications, n (%)** |  |  |  | 0.025 |  |  | <0.001 |
| No complications | 29 (30.5%) | 19 (42.2%) | 10 (20.0%) |  | 24 (48%) | 5 (11.1%) |  |
| Clavien-Dindo grade I | 2 (2.1%) | 2 (4.4%) | 0 (0%) |  | 2 (4%) | 0 (0%) |  |
| Clavien-Dindo grade II | 41 (43.2%) | 19 (42.2%) | 22 (44.0%) |  | 19 (38%) | 22 (48.9%) |  |
| Clavien-Dindo grade III | 12 (12.6%) | 3 (6.7%) | 9 (18.0%) |  | 3 (6%) | 9 (20%) |  |
| Clavien-Dindo grade IV | 8 (8.4%) | 2 (4.4%) | 6 (12.0%) |  | 2 (4%) | 6 (13.3%) |  |
| Clavien-Dindo grade V | 3 (3.2%) | 0 (0%) | 3 (6%) |  | 0 (0%) | 3 (6.7%) |  |
| *Clavien-Dindo > III, n(%)* | 23 (24.2%) | 5 (11.1%) | 18 (36.0%) | 0.005 | 5 (10%) | 18 (40%) | <0.001 |
| Hospital length stay >/= 20 days, n (%) | 42 (44.2 %) | 15 (33.3%) | 27 (54.0%) | 0.043 | 13 (26%) | 29 (64.4%) | 0.000 |
| Mortality, n (%) | 3 (3.2%) | 0 (0%) | 3 (6%) | 0.142 | 0 (0%) | 3 (6.7%) | 0.103 |
|  |  |  |  |  |  |  |  |

*POPF: postoperative pancreatic fistula, CR-IC: Clinically relevant intraabdominal collections, ICU: intensive care unit. *Fisher exact test, otherwise: Chi-square test*
