# Supplementary material for: The role of clinically relevant intra-abdominal collections after pancreaticoduodenectomy: Clinical impact and predictors. A retrospective analysis from a European tertiary centre
Source: Langenbecks Arch Surg. 2023 Dec 28;409(1):21. doi: 10.1007/s00423-023-03200-z (PMC10752846; doi:10.1007/s00423-023-03200-z)
Supplement: Supplementary file 4 — Supplementary file4 (DOCX 20 KB) [file 423_2023_3200_MOESM4_ESM.docx]

**Supplementary Table 4. Sensitivity, specificity, PPV and NPV for clinically relevant intraabdominal collections according to C-reactive protein values at PO3 and PO5.**

|  | **Sensitivity (CI95%)** | | **Specificity (CI95%)** | | **PPV (CI95%)** | | **NPV (CI95%)** | | **AUC values (CI95%)** |
| --- | --- | --- | --- | --- | --- | --- | --- | --- | --- |
| **CRP PO3 > 17.55 mg/dl** | 77.1% | (61%-87%) | 61.7% | (49%-72.9%) | 54% | (40.4%-67%) | 82.2% | (68.7%-90.7%) | 0.731 (0.622-0.84) |
| **CRP PO5 > 13.46 mg/dl** | 82.9% | (67.3%-91.9%) | 73.3% | (61%-82.9%) | 64.4% | (49.8%-76.8%) | 88% | (76.2%-94.4%) | 0.821 (0.736-0.906) |
| *PPV: positive predictive value, NPV: negative predictive value, CI: confidence interval* | | | | | | | | | |
