# Supplementary material for: The role of clinically relevant intra-abdominal collections after pancreaticoduodenectomy: Clinical impact and predictors. A retrospective analysis from a European tertiary centre
Source: Langenbecks Arch Surg. 2023 Dec 28;409(1):21. doi: 10.1007/s00423-023-03200-z (PMC10752846; doi:10.1007/s00423-023-03200-z)
Supplement: Supplementary file 5 — Supplementary file5 (DOCX 21 KB) [file 423_2023_3200_MOESM5_ESM.docx]

**Supplementary Table 5. C-reactive protein median values according to postoperative complications.**

| **A. Complications associating CR-IC and CRP values.** | | | | | | |
| --- | --- | --- | --- | --- | --- | --- |
|  | **CR-POPF** | **Intraabdominal abscess, not related to POPF** | **Bile leak** | **Haemorrhage** | ***p*** |  |
| CRP 3PO mg/dl (mean +/- SD) | 27.4 +/- 9.8 | 24.0 +/- 10.1 | 18.8 +/- 9.0 | 10.4 +/- 9.7 | p=0.000 |  |
| CRP 5PO mg/dl (mean +/- SD) | 22.0 +/- 8.5 | 20.6 +/- 7.5 | 11.3 +/- 8.3 | 14.1 +/- 11.8 | p=0.001 |  |
| **B. CRP values at 3PO and 5PO according to the type of treatment for CR-IC** | | | | | | |
|  | **Surgical drainage** | **Percutaneous drainage** | **Antibiotics alone** | **No treatment** | **p** |  |
| CRP 3PO mg/dl (mean +/- SD) | 26.0 +/-15.8 | 26.6 +/- 5.9 | 23.8 +/- 10.1 | 16.3 +/- 8.9 | p=0.002 |  |
| CRP 5PO mg/dl (mean +/- SD) | 23.8 +/- 14.1 | 18.4 +/- 10.5 | 19.5 +/- 7.2 | 9.6 +/- 7.8 | p=0.000 |  |
| *p=ANOVA test, POPF: postoperative pancreatic fistula, CR-IC: Clinically relevant intraabdominal collections, ICU: intensive care unit. *Fisher exact test, otherwise: Chi-square test* | | | | | |  |
